# Gene Expression Analysis Suggests Immunological Changes of Peripheral Blood Monocytes in the Progression of Patients With Coronary Artery Disease

**DOI:** 10.3389/fgene.2021.641117

**Published:** 2021-03-11

**Authors:** Chunyue Wang, Chenxi Song, Qianqian Liu, Rui Zhang, Rui Fu, Hao Wang, Dong Yin, Weihua Song, Haitao Zhang, Kefei Dou

**Affiliations:** Fuwai Hospital, National Center for Cardiovascular Diseases, State Key Laboratory of Cardiovascular Disease, Chinese Academy of Medical Sciences and Peking Union Medical College, Beijing, China

**Keywords:** coronary artery disease, intermediate coronary lesions, acute myocardial infarction, RNA sequencing, differentially expressed genes

## Abstract

**Objectives:**

To analyze the gene expression profile of peripheral blood monocytes in different stages of coronary artery disease (CAD) by transcriptome sequencing, and to explore potential genes and pathway involved in CAD pathogenesis.

**Methods:**

According to the screening of coronary angiography and quality control of blood samples, eight intermediate coronary lesion patients were selected, then eight patients with acute myocardial infarction, and eight patients with normal coronary angiography were matched by age and gender. Transcriptomics sequencing was conducted for the peripheral blood monocytes of these 24 samples by using the Illumina HiSeq high-throughput platform. Then, differentially expressed genes (DEGs) were analyzed. Gene Ontology (GO) functional annotation, Kyoto Encyclopedia of Genes and Genomes (KEGG) pathway annotation, and protein-protein interaction (PPI) network were applied to annotate the potential functions of DEGs.

**Results:**

Compared with the normal coronary angiography group, we identified a total of 169 DEGs in the intermediate coronary lesion group, which were significantly enriched in 59 GO terms and 17 KEGG pathways. Compared with the normal coronary angiography group, we found a total of 2,028 DEGs, which were significantly enriched in 311 GO terms and 20 KEGG pathways in the acute myocardial infarction group. The cross-comparison between normal versus intermediate coronary lesion group, and normal versus acute myocardial infarction group included 98 differential genes with 65 up regulated and 33 down regulated genes, which were significantly enriched in 46 GO terms and 10 KEGG pathways. During the progression of CAD, there was a significant up-regulated expression of CSF3, IL-1A, CCR7, and IL-18, and down-regulated expression of MAPK14. Besides GO items such as inflammatory response was significantly enriched, KEGG analysis showed the most remarkable enrichments in IL-17 signaling pathway and cytokine-cytokine receptor interactions.

**Conclusions:**

Transcriptomics profiles vary in patients with different severity of CAD. CSF3, IL-1A, CCR7, IL-18, and MAPK14, as well as IL-17 signaling pathway and cytokine and cytokine receptor interaction signaling pathway related with inflammatory response might be the potential biomarker and targets for the treatment of coronary artery disease.

## Introduction

The immune system plays a pivotal role in the pathogenesis of the initiation and progression of coronary atherosclerosis and coronary artery disease (CAD) (Christodoulidis et al., 2014). Since peripheral blood monocytes play multiple key roles in the immune system, screening gene expression changes of monocytes might be helpful to explore new diagnostic markers and therapeutic targets for CAD. In this study, we establish gene expression profiling of peripheral blood monocytes in the development of CAD, and identify the differentially expressed genes (DEGs) associated with the different severity of CAD by high-throughput sequencing techniques. Combined with bioinformatics analysis, we illustrated the functions of DEGs and identified novel biomarkers that are potentially involved in CAD pathogenesis.

Inflammation caused by immune system plays an important role in the pathophysiology of coronary heart disease (Cavieres, 2020). Role of inflammatory mediators was highlighted in the current understanding of the development of CAD (Gidron et al., 2007). Overexpression of miR146a, an inflammatory mediator, reduced cardiac functional abnormalities with decreased inflammatory markers levels (Feng et al., 2017). Various evidences indicated that inflammation related cytokines and chemokines were involved in the initiation and progression of CAD, such as IL-2, IL-6, IL-9, IL-10, IL-17, TNF-a, IFN-g (Hashmi and Zeng, 2006; Lu et al., 2013; Al Shahi et al., 2015; Tang et al., 2015; Zhu et al., 2015; Min et al., 2017). IL-1 signaling was regarded as an essential inducer in the pathogenesis of heart failure because it increases cardiomyocyte apoptosis and inhibits cardiac contractility (Bujak and Frangogiannis, 2009). IL-17 level in serum was used to represent the inflammation levels in CAD patients. Down-regulation of IL-17 and TNF-a levels could be associated with decreased symptoms in a CAD rat model (Fang et al., 2018). Genetic analysis showed that some SNPs such as IL-17 and IL-23 gene polymorphisms were highly associated with risk of CAD (Shuang et al., 2015; Zhao et al., 2019). These works indicated that CAD was a complicated disease with high immunological and hereditary correlation.

## Materials and Methods

### Patient Selection

The diagnosis was made on the basis of symptoms, laboratory tests, electrocardiogram (ECG), and coronary angiographic results. Patients with no stenosis in coronary arteries comprised the normal coronary artery (NCA) group. Intermediate coronary lesion (ICL) group was defined angiographically as 50−70% luminal diameter obstruction in at least one major coronary artery vessel. The diagnostic criteria for acute myocardial infarction (AMI) group was based on the WHO classification of disease. Patients of AMI group had ischemic chest pain, increased values of cardiac enzymes, and dynamic ST-T change on a surface ECG, meanwhile luminal diameter obstruction up to 80−100%. Coronary angiography images were assessed by two interventional physicians blindly. We excluded patients with aortic dissection, pulmonary embolism, malignant tumor, autoimmune disorders, severe infectious diseases, trauma, severe heart failure with left ventricular ejection fraction <20%, liver dysfunction (alanine aminotransferase level > 135 U/l), severe renal dysfunction (creatinine > 3.0 mg/dl), or blood-borne infectious diseases, including human immunodeficiency virus/acquired immunodeficiency syndrome, hepatitis B, and hepatitis C. We also excluded patients with myocarditis, pericarditis, and Takotsubo cardiomyopathy. Afterward, according to the screening of coronary angiography and quality control of blood samples, eight patients who underwent coronary angiography to evaluate CAD at Fuwai Hospital between May 2013 and March 2015 were collected for our study to form ICL group. Then, eight patients with acute myocardial infarction and eight patients with normal coronary angiography were matched by age and gender. The final cohort included 24 patients with different severity of CAD. Informed consent was obtained from all patients. The Ethics Committee of Fuwai Hospital approved this study, which complies with the Declaration of Helsinki.

### RNA Extraction, Construction of cDNA Library, and Transcriptome Sequencing

Total RNA was extracted from peripheral blood monocyte separated from blood samples within 24 h after hospitalized using TRIzol reagent (Invitrogen). The isolated RNA with qualified purity and integrity was reversely transcribed into the first strand cDNA with OligodT nucleic acid sequence. After further amplification, enrichment, and purification, the cDNA library was constructed, and double terminal 2 × 150 bp sequencing was performed on Illumina high-throughput sequencing platforms (San Diego, CA, United States).

### Transcriptome Data Quality Control and Comparison

The original sequences were filtered by the Cutadapt software^1^ (Martin, 2011) to obtain the high quality sequences (clean reads) with base mass value Q30 of more than 95% and GC of more than 40%. Then, the human genome sequences and annotations were downloaded from the genome sequence database ENSEMBL (version Homo_sapiens.GRCh38.91.chr)^2^. The clean reads were mapped to the human reference genome using the HISAT2 software (version 2.1.0) (Kim et al., 2015).

### Identification of Differentially Expression Genes (DEGs)

HTSeq software (version 0.6.0) (Anders et al., 2015) was used to calculate the gene count, and Fragments per Kilobase per Million Mapped Fragments (FPKM) (Trapnell et al., 2010) was carried out to convert the count number to eliminate the influence of gene expression caused by gene length and sequencing quantity. The DESeq2 (version 1.20.0) (Love et al., 2014) package was used to analyze the differential expression of genes and to calculate the *P*-value between each pair of groups. Furthermore, to avoid false positive results due to multi-test problems, the raw *P*-values were adjusted into the false discovery rate (FDR) using the Benjamin and Hochberg method. The combined threshold values of differentially expressed genes (DEGs) from each pair of groups were all set as FDR < 0.05 and | log2 fold change (FC)| ≥ 1. On account of the results of FPKM of DEGs, the volcano plot and heat map were drawn by using the “ggplot2”and “pheatmap” packages in R.

### Functional Enrichment Analysis of DEGs

Studies on large-scale transcription data or genomic data were usually performed based on Gene Ontology (GO) analyses (Chan et al., 2019). The Kyoto Encyclopedia of Genes and Genomes (KEGG) pathway database harbors information relating to the networks among genes or molecules, which was used for genetic studies (Kanehisa and Goto, 2000; Kanehisa et al., 2014). The differentially expressed genes were annotated by the GO and KEGG database. The online tool Database for Annotation, Visualization and Integrated Discovery (DAVID) (version 6.8; david. abcc.ncifcrf.gov) and METASCAPE^3^ (Zhou et al., 2019) were used to detect gene ontology categories and KEGG pathways. *P*-value < 0.05 was defined as the threshold for significant enrichment of GO and KEGG analyses. Furthermore, to gain insights into the interaction between proteins encoded by DEGs, a protein-protein interaction (PPI) network of DEGs was constructed with the Search Tool for the Retrieval of Interacting Genes database (STRING)^4^. To assist visualizing the biological networks and integrating the data generated by the STRING database, the PPI networks were visualized using the Cytoscape (version 3.7.2^5^) (Cline et al., 2007). Combined scores greater than 0.4 were considered statistically significant and the hub genes in this PPI network were extracted by using the cytoHubba application. A node represents a gene; the undirected link between two nodes is an edge, denoting the interaction between two genes. Hub genes, involved in much more interactions, were highly connected nodes, which may be more important than other genes in the whole network. The degree of a node corresponds to the number of interactions of a gene with other genes. The significance of gene nodes in the network was described using a connectivity degree.

### Statistical Analysis

Continuous data were expressed as mean ± SD and compared by using ANOVA. Categorical variables were presented as counts (frequency) and compared using chi-square test or the Fisher exact test, as appropriate. A two-sided *P* < 0.05 was considered to statistical significant. Statistical analysis were performed by using SAS 9.4 (SAS Institute, Cary, NC, United States).

## Results

### Clinical Characteristics of the Patients Recruited

The baseline characteristics of the eight ICL cases and eight age-and gender-matched NCA and AMI cases were displayed in Table 1. Among the 24 patients, 17 persons (70.83%) were male, with a median age of 64 years (interquartile range: 59.5−67.5 years). Additionally, there was no significant difference in age, gender, and body mass index (BMI) among the different groups. As expected, CAD patients had significantly higher rate of hyperlipidemia and family history of CAD and more likely to be current smoker, but the different did not achieve statistical significance.

**TABLE 1:** Comparison baseline demographic data of patients (the values are presented as mean ± SD or as percentages).

	Normal Control (*n* = 8)	Intermediate Lesion (*n* = 8)	Myocardial Infarction (*n* = 8)	*P* value
Male (%)	5/8 (62.50)	6/8 (75.00)	6/8 (75.00)	1.000
Age (year)	59.75 ± 8.71	63.38 ± 9.04	63.25 ± 8.29	0.741
Body mass index	24.75 ± 2.13	22.72 ± 2.28	26.00 ± 3.31	0.062
Diabetes mellitus(%)	2/8 (25.00)	1/8 (12.50)	3/8 (37.50)	0.837
Hypertension(%)	6/8 (75.00)	5/8 (62.50)	4/8 (50.00)	0.866
Hyperlipidemia(%)	5/8 (62.50)	5/8 (62.50)	7/8 (87.50)	0.866
Family history of coronary artery disease(%)	0/8 (0.00)	7/8 (87.50)	1/8 (12.50)	0.557
Smoking(%)	1/8 (12.50)	5/8 (62.50)	3/8 (37.50)	0.171
Alcoholic(%)	4/8 (50.00)	1/8 (12.50)	2/8 (25.00)	0.402

### Transcriptome Sequencing Results and Annotations

According to the transcriptome sequencing, 56,816,018, 58,878,194, and 56,215,122 sequences were obtained from the NCA, ICL, and AMI group, respectively. Besides, the bases of three groups were 8,499,984,489, 8,813,036,038, and 8,418,443,960 bp. The mapping rate of all the samples ranged from 96.27 to 98.73%, with Q30 > 95% for each sample. Compared with the reference genome, the average mapping rates of the three groups were 93.06, 91.57, and 94.31%. Generally, these results show that the quality of these libraries was good and suitable for analysis. After all a total of 49,527 genes were annotated, and their FPKM values in 24 samples were shown in Supplementary Table 1. Microarray data have been submitted to the GEO repository under accession number GSE166780.

### Different Expression Genes Analysis of Different Severity of CAD Patients

**FIGURE 1:**
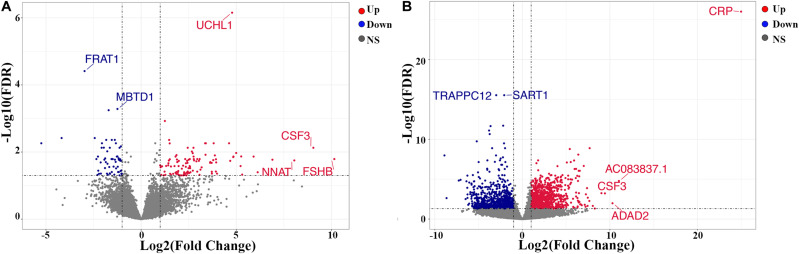
Volcano plots of differentially expressed genes (DEGs). **(A)** ICL group vs. NCA group; **(B)** AMI group vs. NCA group. *X*-axis represents log2 the fold change of gene expression in the compared groups, and *Y*-axis represents-1 × log10 (FDR value) for each DEG. Red dots indicate up-regulated DEGs, blue dots indicate down-regulated DEGs, and gray dots indicate non-significant DEGs. DEGs, differentially expressed genes. NS, None Significant.

**FIGURE 2:**
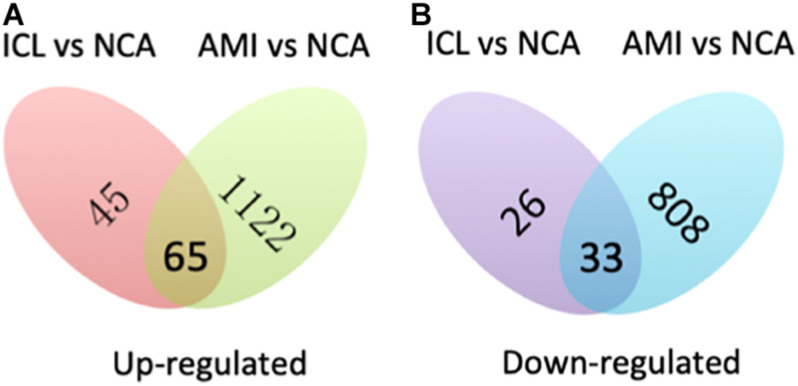
Venn map shows the intersection of up-regulated **(A)** and down-regulated **(B)** DEGs between ICL vs. NCA and AMI vs. NCA.

**FIGURE 3:**
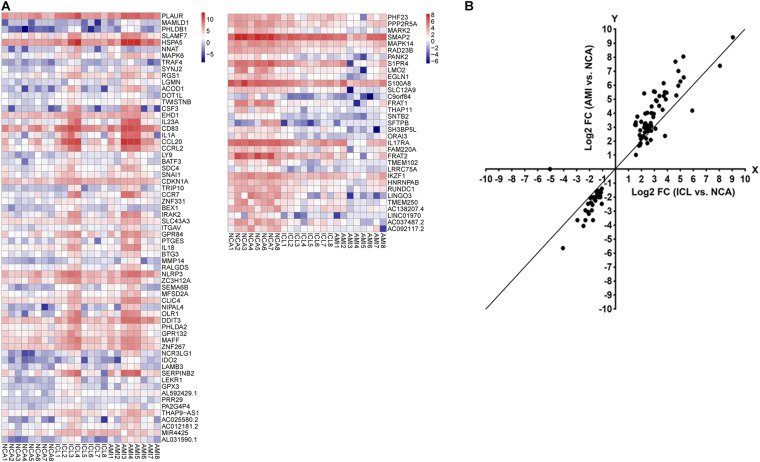
**(A)** The heat map of the 98 DEGs overlapping between ICL group vs. NCA group and AMI group vs. NCA group. The *X*-axis represents the samples, NCA1 – NCA8: NCA controls (*n* = 8); ICL1 – ICL8: ICL patients (*n* = 8); AMI1 – AMI8: AMI patients (*n* = 8). The *Y*-axis denotes the DEGs. The red and blue tones in the picture indicate the expression levels of DEGs (represents by log_2_FPKM) and the colors changing from blue to red indicate higher expression levels. **(B)** The scatter plot of the 98 DEGs. The *X*-axis represents the log2 FC of DEGs between ICL and NCA. The *Y*-axis denotes log2 FC of DEGs between AMI and NCA. DEGs, differentially expressed genes; FPKM, Fragments per Kilobase per Million Mapped Fragments; FC, Fold Change.

A total of 169 genes, including 110 up-regulated genes and 59 down-regulated genes, were defined as DEGs in comparison between the ICL group and NCA group (Supplementary Table 1) with thresholds of | log2 FC| ≥ 1 and FDR < 0.05. Compared with the NCA group, we identified 2,028 DEGs consisting of 1,187 up-regulated and 841 down-regulated in the AMI group (Supplementary Table 2). Analysis of the volcano plot and heatmap of hierarchical clustering showed that the identified DEGs could easily distinguish patients with CAD from normal controls (Figure 1 and Supplementary Figure 1). The top six marker genes were selected according to the rank of fold change and FDR value, respectively. Between ICL and NCA, based on | log2 FC| ≥ 1, the top three DEGs with the lowest FDR are UCHL1, FRAT1, and MBTD1; while meeting the standard of FDR < 0.05, the top three DEGs with the highest | log2 FC| are FSHB, CSF3, and NNAT. While compared to NCA, the top three regulated marker genes with the lowest FDR or the highest | log2 FC| in AMI are: CRP, SART1, TRAPPC12, and AC083837.1, ADAD2, CSF3, respectively. The gene expression levels in different groups were showed in Supplementary Figures 2A–K. As coronary stenosis exists in both the ICL group and the AMI group, we explored the overlapping differential genes between NCA versus ICL group and NCA versus AMI group in order to find genes involved in the development of CAD. A total of 98 genes were identified to be regulated consistently, which included 65 up regulated and 33 down regulated (Supplementary Table 3 and Figure 2). Figure 3 is the heat map and scatter plot showing the expression levels of the 98 DEGs of each individual, from which we can clearly see that the trend of most common DEGs’ fold change is consistent with the severity of CAD.

### GO Functional Enrichment Analysis of DEGs Across Different Groups

**FIGURE 4:**
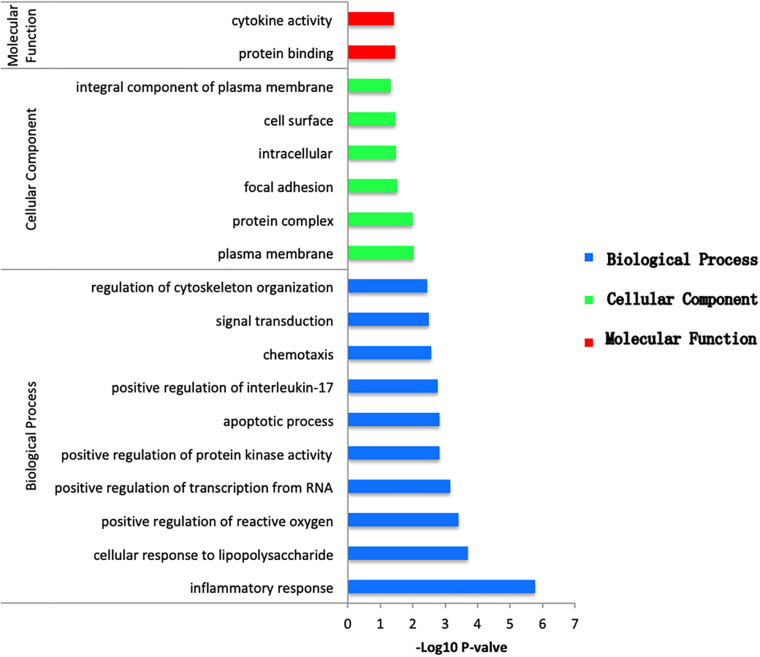
Bar plot of GO enrichment analysis of DEGs overlapping between ICL group vs. NCA group and AMI group vs. NCA group. *X*-axis represents –log10 *P* value. *Y*-axis represents different functional groups (also named as different GO terms). The green bars indicate cellular component terms, the blue bars indicate biological process terms, and the red bars indicate molecular function term.

To obtain the biological function of DEGs, the online tool DAVID was used to predict GO categories and enrichment. GO functional enrichment analysis was categorized in terms of biological processes (BP), cellular components (CC), and molecular functions (MF). According to the following criteria: *P* value < 0.05, between the NCA and ICL, 59 items were enriched, including 46 BP, 6 CC, and 7 MF (Supplementary Figure 3). The DEGs were mostly enriched in secretory granule and involved in protein binding of molecular function and inflammatory response of biological processes. Between the NCA and AMI, 311 items were enriched, including 222 BP, 47 CC, and 42 MF (Supplementary Figure 4). Among which cellular response to lipopolysaccharide was the most significant enriched items in BP. In addition, nucleoplasm was considered as the most remarkably enriched items in CC. In the MF category, protein binding showed significant enrichment. Finally, with thresholds of *P* < 0.05, a total of 46 GO items including 38 BP, 6 CC, and 2 MF were significantly enriched in the 98 overlapping genes selected from integrative analysis of between NCA versus ICL group and NCA versus AMI group. Based on the *P* values, the GO terms of the three categories were shown in Figure 4. The GO functional enrichment of these potential DEGs showed that the inflammation response in BP category was identified as the highest enrichment, while in the MF category protein binding and in the CC category nucleoplasm showed remarkable enrichment.

### KEGG Pathways Analysis of DEGs Across Different Groups

**FIGURE 5:**
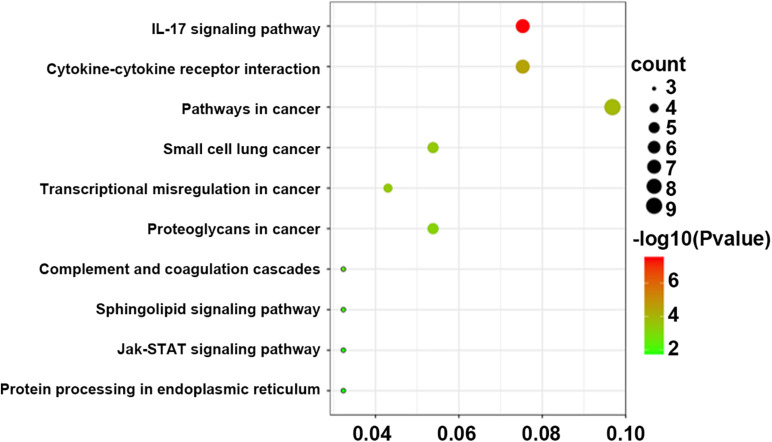
Bubble Plot of KEGG enrichment analysis of DEGs overlapping between ICL group vs. NCA group and AMI group vs. NCA group. *X*-axis represents ratios of the number of differential genes to the total gene number in a specific pathway. The color and size of the dots represent significance and amount of genes enrichment, respectively. DEGs, differentially expressed genes, KEGG, Kyoto Encyclopedia of Genes and Genomes.

**FIGURE 6:**
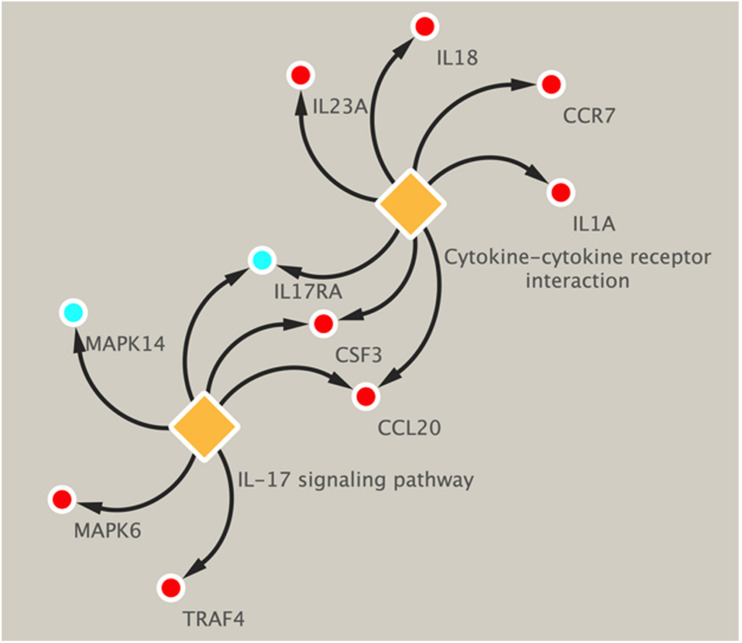
KEGG enrichment pathways of DEGs related to IL-17 signal pathway and cytokine–cytokine receptor interaction. Up-regulated genes are marked with red, down-regulated genes are marked with blue. DEGs, differentially expressed genes; KEGG, Kyoto Encyclopedia of Genes and Genomes.

Pathway analysis was utilized to find the significant pathways of the DEGs according to the KEGG database. In our study, the KEGG pathway enrichment analysis was implemented by the online tool Metascape (see text footnote 2). A *P* value < 0.05 was used as the cut-off criterion to identify KEGG enrichment pathways. The 169 DEGs between the NCA and ICL were significantly associated with 17 KEGG pathways and 2,028 DEGs between the NCA and AMI were enriched in 20 KEGG pathways (Supplementary Figure 5). The 98 overlapping DEGs of the cross-comparison between NCA versus ICL group and NCA versus AMI group were annotated by the KEGG database and the ten signaling pathways are illustrated in Figure 5. Furthermore, the top two KEGG pathways were identified based on FDR < 0.05, including IL-17 signaling pathway and cytokine-cytokine receptor interaction pathway (Figure 6).

### PPI Analysis of the Overlapping DEGs Identified by Cross-Comparison

**FIGURE 7:**
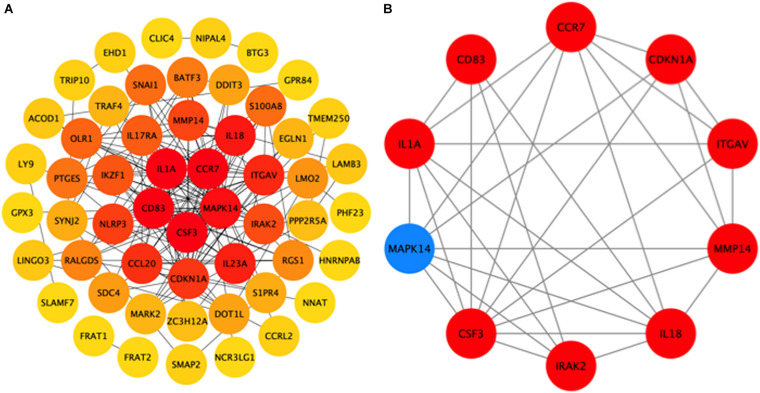
Hub genes identified from the protein-protein interaction (PPI) network. **(A)** PPI network of 98 DEGs related to the progression of CAD, consisting of 54 nodes and 161 edges. The edge indicates the interaction between two genes. A degree is used to describe the importance of the protein nodes in the network. Color of the hubs is related to connectivity degree. Brighter red of node color is for more connectivity of the DEGs. **(B)** PPI network of 10 hub genes extracted from **(A)**. Up-regulated genes are marked with red, down-regulated genes are marked with blue. CAD, coronary artery disease. DEGs, differentially expressed genes.

**FIGURE 8:**
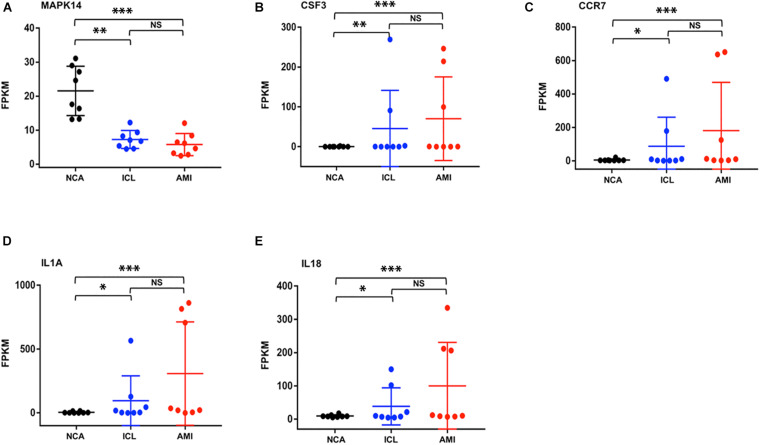
The expression levels of five hub genes (represents by FPKM) identified from the PPI analysis **(A–E)**. *P < 0.05, **P < 0.01, ***P < 0.001.

To further explore the interactions between the 98 DEGs of the cross-comparison between NCA versus ICL group and NCA versus AMI group, a PPI network was constructed including 54 nodes and 161 edges (Figure 7A). By using the cytoHubba plugin for Cytoscape, genes with connectivity degree above 10 in PPI network analysis were screened as hub genes significantly associated with CAD. The ten most significant genes were MAPK14, CDKN1A, CCR7, CSF3, IL1A, ITGAV, MMP14, IL18, CD83, and IRAK2 (Figure 7B). The network distances of ten hub genes were all 1. Among them there were five genes enriched in IL-17 signaling pathway and cytokine-cytokine receptor interaction pathway that were the significant enriched pathway of the 98 overlapping genes described above. These five genes included four up-regulated genes: CSF3, degree = 18; IL-1A, degree = 14; CCR7 degree = 18; IL-18, degree = 13; and one down-regulated gene: MAPK14, degree = 25. The expression levels of these five hub genes identified from the PPI analysis are displayed in Figures 8A–E.

## Discussion

Previous studies have shown the gene expression of peripheral blood monocytes may be involved in the formation and development of atherosclerosis and CAD. But most studies concentrate on patients in normal and stenosis coronary artery (Sinnaeve et al., 2009; Liu et al., 2018), or patients with acute myocardial infarction and stable CAD (Kiliszek et al., 2012; Holvoet et al., 2019), which demonstrate little evidence of gradual progression of CAD. In this study, we analyze and compare the gene expression of peripheral blood monocytes in normal coronary artery, intermediate lesion, and myocardial infarction patients with high-throughput sequencing technique. Compared with the NCA group, the DEGs in the other two groups indicate the genes associated with the formation of atherosclerosis and CAD, and the overlapping of the two groups can be regarded as internal validation for more reliability. Meanwhile, normal control, intermediate lesion, and myocardial infarction represent the onset and gradual progression stages of CAD. It is also helpful to confirm the genes involved progression of CAD with analyzing the DEGs among different groups.

Gene ontology terms include cellular component (CC), molecular function (MF), and biological process (BP). Between the NCA and ICL group, there was only six items significant enrichment in CC and seven items in MF while there were more enrichment in BP, which may suggest cellular component and molecular function have no significant changing in early stage of CAD. Meanwhile between the NCA and AMI group, there were 47 GO items enrichment in CC and 42 items in MF, which implying cellular component and molecular function may play an important role in the progression and repairment of injured myocardium. This interesting finding suggested that, further study on the DEGs enriched in CC and MF parts of go items is more valuable for finding intervention targets of AMI. While DEGs enriched in the GO items of BP categories are more useful to explore the early diagnosis and therapeutic targets of CAD.

Further analyses with KEGG database and PPI network, confirm the inflammatory response, signaling pathway of IL-17, and cytokine-cytokine receptor interaction as the identified DEGs’ main functions of peripheral blood monocytes in the progression of CAD patients. Enrichment analysis of GO terms and KEGG pathways related with DEGs provide several known and novel molecular mechanisms associated with the initiation and progression of CAD. The hub genes with high connectivity degree include MAPK14, CSF3, CCR7, IL-1A, and IL-18. The proteins encoded by the genes above belong to mitogen activated protein kinase (MAPK), colony stimulating factor (CSF), chemokine receptors (CCR), and interleukin (IL) superfamily, respectively. MAPK signal pathway is one of the core components of cell stress, inflammation, differentiation, and apoptosis. The activation of MAPK is the final step of intracellular phosphorylation cascade reaction. Previous studies reported that MAPK14 is implicated in the pathogenesis of many inflammatory-driven conditions, including atherosclerosis (Fisk et al., 2014). The increased expression of CSF3 gene in smooth muscle phenotype transition is one of the important mechanisms of atherosclerosis (Karagiannis et al., 2013). CCR7 knockout can inhibit the formation of atherosclerotic plaque in mice (Luchtefeld et al., 2010), while it significantly increases in patients with carotid and coronary atherosclerosis, to promote monocyte adhesion and migration (Cai et al., 2014). IL-1A encodes IL-1α protein, both bone marrow cell knockout of IL-1A (Kamari et al., 2011) and application of IL-1α specific antibody (Tissot et al., 2013) can inhibit the formation of atherosclerotic plaque in mice. IL-18 shows the similar characteristic in model study (Elhage et al., 2003). KEGG enrichment pathways showed that some inflammatory cytokines signal pathways and chemokines interaction such as IL-1, IL-17, IL-23, and CCL20, CCR7 were highly associated with the progression of CAD. This indicated inflammatory immune response might be a key regulatory link in the initiation and development of CAD.

Limitations: in recent years, dozens of reports have explored the diagnostic value of DEGs in atherosclerosis and CAD, however, the results are inconsistent due to the various platforms and small sample number. There is similar shortcoming in this observational case controlled study with limited subjects. Furthermore, although several DEGs have been identified, the exact relationship between the DEGs and the pathophysiological progression of CAD is still uncertain. Functional validation was lacked for the feature genes obtained here. Further investigations for these identified genes are required with substantial experiments. Based on the detected DEGs, to explore the transcription and protein expression, as well as the regulation for these, DEGs in the onset and course of progression of CAD will be carried out and verified in the coming future.

In conclusion, this study established the gene expression profile of peripheral blood monocyte in different stages of CAD patients successfully. Moreover, our reports suggest that gene CSF3, IL-1A, CCR7, IL-18, MAPK14, as well as IL-17 signaling pathway and cytokine- cytokine receptor interaction pathway, may serve as the potential diagnostic or prognostic gene markers and therapeutic targets for CAD.

## Data Availability Statement

The datasets presented in this study can be found in online repositories. Microarray data have been submitted to the GEO repository under accession number GSE166780. The names of the repository/repositories and accession number(s) can be found in the article/Supplementary Material.

## Ethics Statement

The studies involving human participants were reviewed and approved by the Ethics Committee of Fuwai Hospital. The patients/participants provided their written informed consent to participate in this study. Written informed consent was obtained from the individual(s) for the publication of any potentially identifiable images or data included in this article.

## Author Contributions

KD and HZ contributed equally to conception and design of the study. CW contributed in writing the manuscript. WS and DY revised the manuscript critically for important intellectual content. QL and RF were in charge of screening patients and inquiring about medical records. DY and HW assessed coronary angiogram blindly. CS and RZ contributed to analysis and interpretation of data. All authors contributed to the article and approved the submitted version.

## Conflict of Interest

The authors declare that the research was conducted in the absence of any commercial or financial relationships that could be construed as a potential conflict of interest.

## Footnotes

1 http://pypi.python.org/pypi/cutadapt

2 http://www.ensembl.org

3 http://metascape.org

4 http://string-db.org/

5 http://cytoscape.org/

## Supplementary Material

Supplementary Figure 1 Hierarchical clustering of DEGs in the different stages of CAD patients and NCA controls. **(A)** ICL group vs. NCA group; **(B)** AMI group vs. NCA group. The *X*-axis represents the samples, NCA1-NCA8: NCA controls (*n* = 8); ICL1−ICL8: ICL patients (*n* = 8); AMI1−AMI8: AMI patients (*n* = 8). The *Y*-axis denotes the DEGs. The red and blue tones in the picture indicate the expression levels of DEGs (represents by log2 FPKM) and the colors changing from blue to red indicate higher expression levels. DEGs, differentially expressed genes; FPKM, Fragments per Kilobase per Million Mapped Fragments.

Supplementary Figure 2 The expression levels of the marker genes (represents by FPKM) identified from the Volcano plot of differentially expressed genes(**A–K)**. ^∗^*P* < 0.05, ^∗∗^*P* < 0.01, ^∗∗∗^*P* < 0.001.

Supplementary Figure 3 GO enrichment analysis of DEGs between ICL group and NCA group. **(A)** Bubble Plot of BP. **(B)** Bubble Plot of CC. **(C)** Bubble Plot of MF. *X*-axis represents ratios of the number of differential genes to the total gene number in a specific pathway. *Y*-axis represents different functional groups (also named as different GO terms). The color and size of the dots represent significance and amount of genes enrichment, respectively. DEGs, differentially expressed genes; GO, Gene Ontology.

Supplementary Figure 4 GO enrichment analysis of DEGs between AMI group and NCA group. **(A)** Bubble Plot of BP. **(B)** Bubble Plot of CC. **(C)** Bubble Plot of MF. *X*-axis represents ratios of the number of differential genes to the total gene number in a specific pathway. *Y*-axis represents different functional groups (also named as different GO terms). The color and size of the dots represent significance and amount of genes enrichment, respectively. DEGs, differentially expressed genes; GO, Gene Ontology.

Supplementary Figure 5 KEGG enrichment analysis of DEGs between ICL group vs. NCA group and AMI group vs. NCA group. **(A)** ICL vs. NCA. **(B)** AMI vs. NCA. *X*-axis represents ratios of the number of differential genes to the total gene number in a specific pathway. *Y*-axis represents different KEGG pathways. The color and size of the dots represent significance and amount of genes enrichment, respectively. DEGs, differentially expressed genes; KEGG, Kyoto Encyclopedia of Genes and Genomes.

Supplementary Table 1 FPKM of 49527 genes in 24 samples.

Supplementary Table 2 169 differentially expressed genes between ICL and NCA.

Supplementary Table 3 2,028 differentially expressed genes between AMI and NCA.

Supplementary Table 4 98 differentially expressed genes in different stages of coronary artery disease patients.
