# Supplementary figures and images for: Gene Expression Analysis Suggests Immunological Changes of Peripheral Blood Monocytes in the Progression of Patients With Coronary Artery Disease
Source: Front Genet. 2021 Mar 11;12:641117. doi: 10.3389/fgene.2021.641117 (PMC7990797; doi:10.3389/fgene.2021.641117)

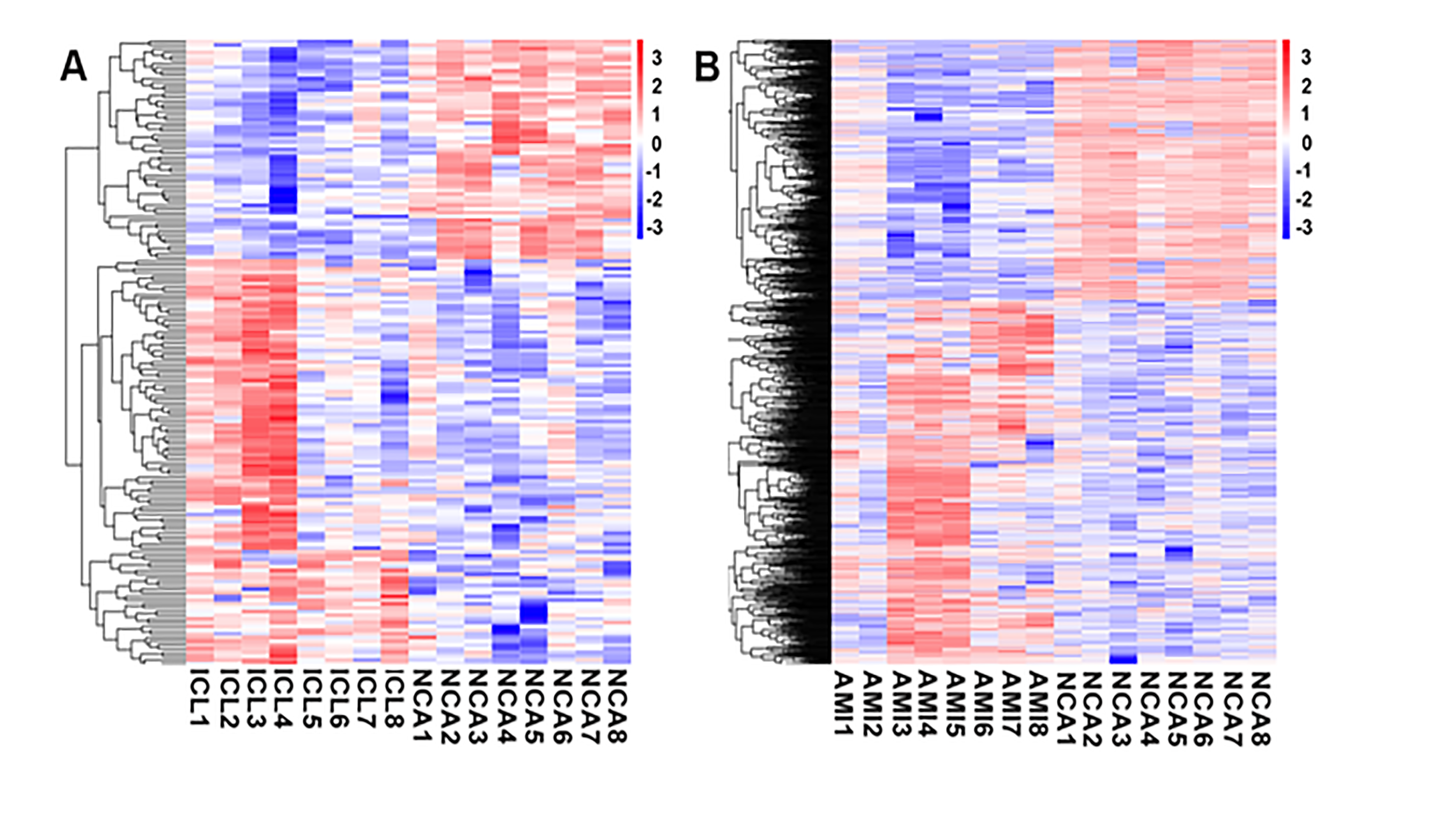

Supplement: Supplementary Figure 1 — Hierarchical clustering of DEGs in the different stages of CAD patients and NCA controls. (A) ICL group vs. NCA group; (B) AMI group vs. NCA group. The X-axis represents the samples, NCA1-NCA8: NCA controls (n = 8); ICL1−ICL8: ICL patients (n = 8); AMI1−AMI8: AMI patients (n = 8). The Y-axis denotes the DEGs. The red and blue tones in the picture indicate the expression levels of DEGs (represents by log2 FPKM) and the colors changing from blue to red indicate higher expression levels. DEGs, differentially expressed genes; FPKM, Fragments per Kilobase per Million Mapped Fragments. [file Image_1.TIF]

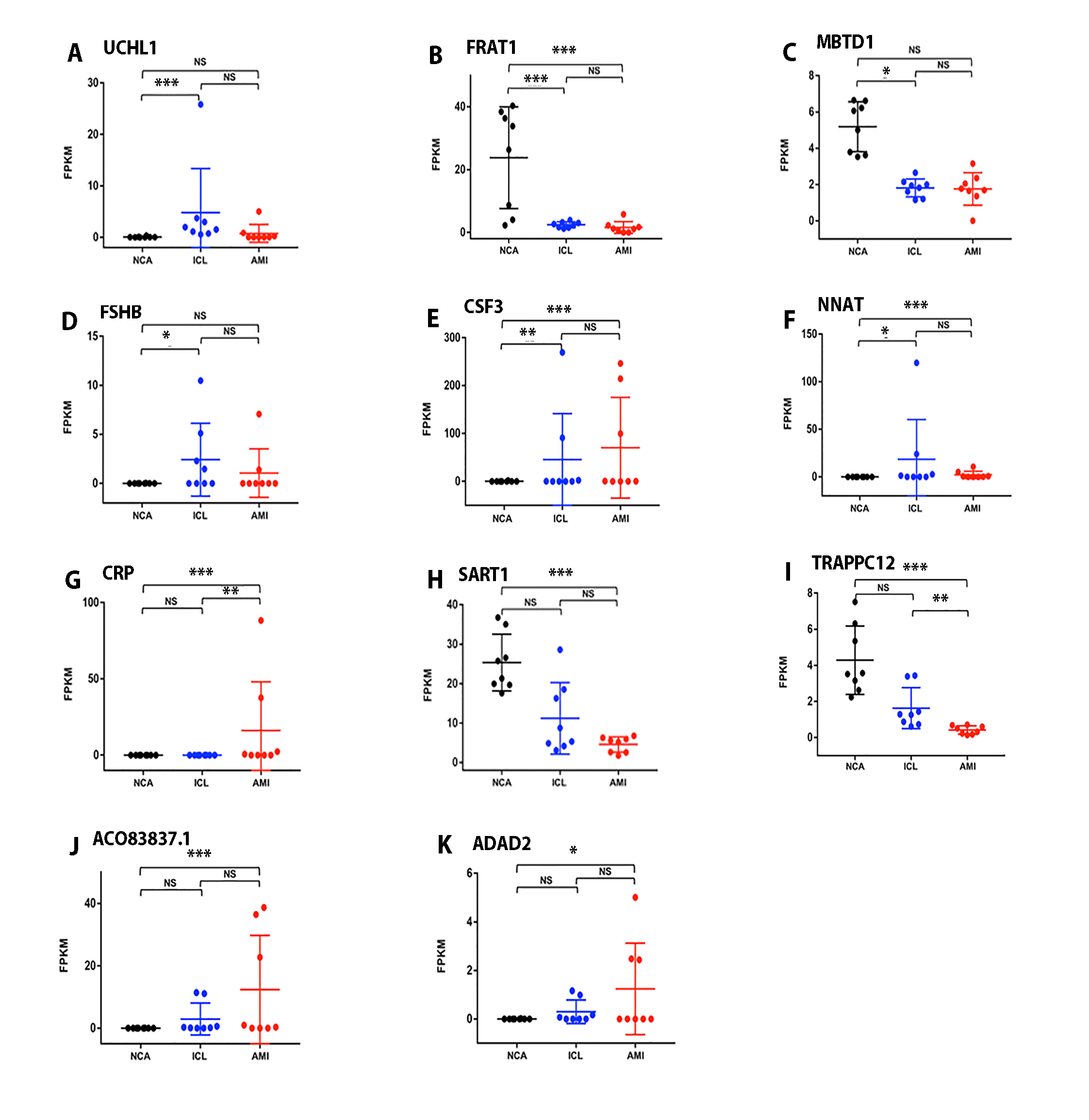

Supplement: Supplementary Figure 2 — The expression levels of the marker genes (represents by FPKM) identified from the Volcano plot of differentially expressed genes(A–K). ∗P < 0.05, ∗∗P < 0.01, ∗∗∗P < 0.001. [file Image_2.TIF]

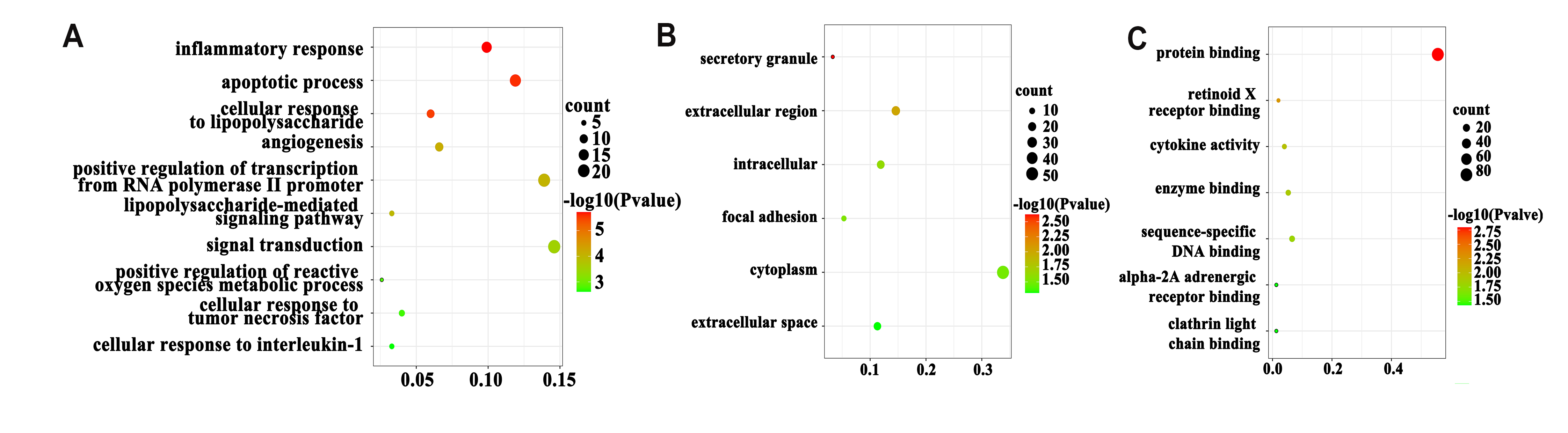

Supplement: Supplementary Figure 3 — GO enrichment analysis of DEGs between ICL group and NCA group. (A) Bubble Plot of BP. (B) Bubble Plot of CC. (C) Bubble Plot of MF. X-axis represents ratios of the number of differential genes to the total gene number in a specific pathway. Y-axis represents different functional groups (also named as different GO terms). The color and size of the dots represent significance and amount of genes enrichment, respectively. DEGs, differentially expressed genes; GO, Gene Ontology. [file Image_3.TIF]

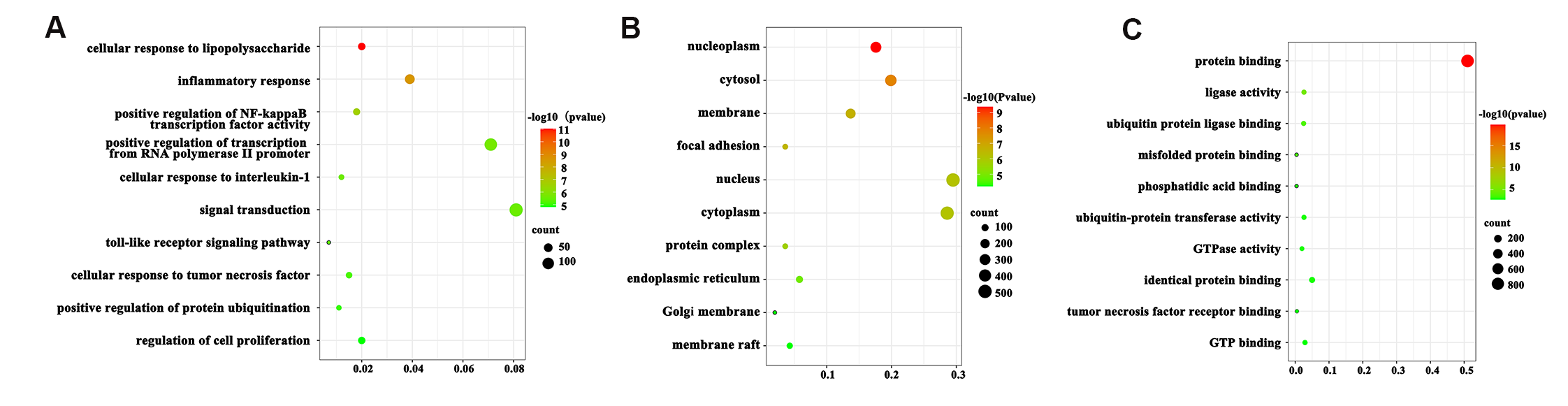

Supplement: Supplementary Figure 4 — GO enrichment analysis of DEGs between AMI group and NCA group. (A) Bubble Plot of BP. (B) Bubble Plot of CC. (C) Bubble Plot of MF. X-axis represents ratios of the number of differential genes to the total gene number in a specific pathway. Y-axis represents different functional groups (also named as different GO terms). The color and size of the dots represent significance and amount of genes enrichment, respectively. DEGs, differentially expressed genes; GO, Gene Ontology. [file Image_4.TIF]

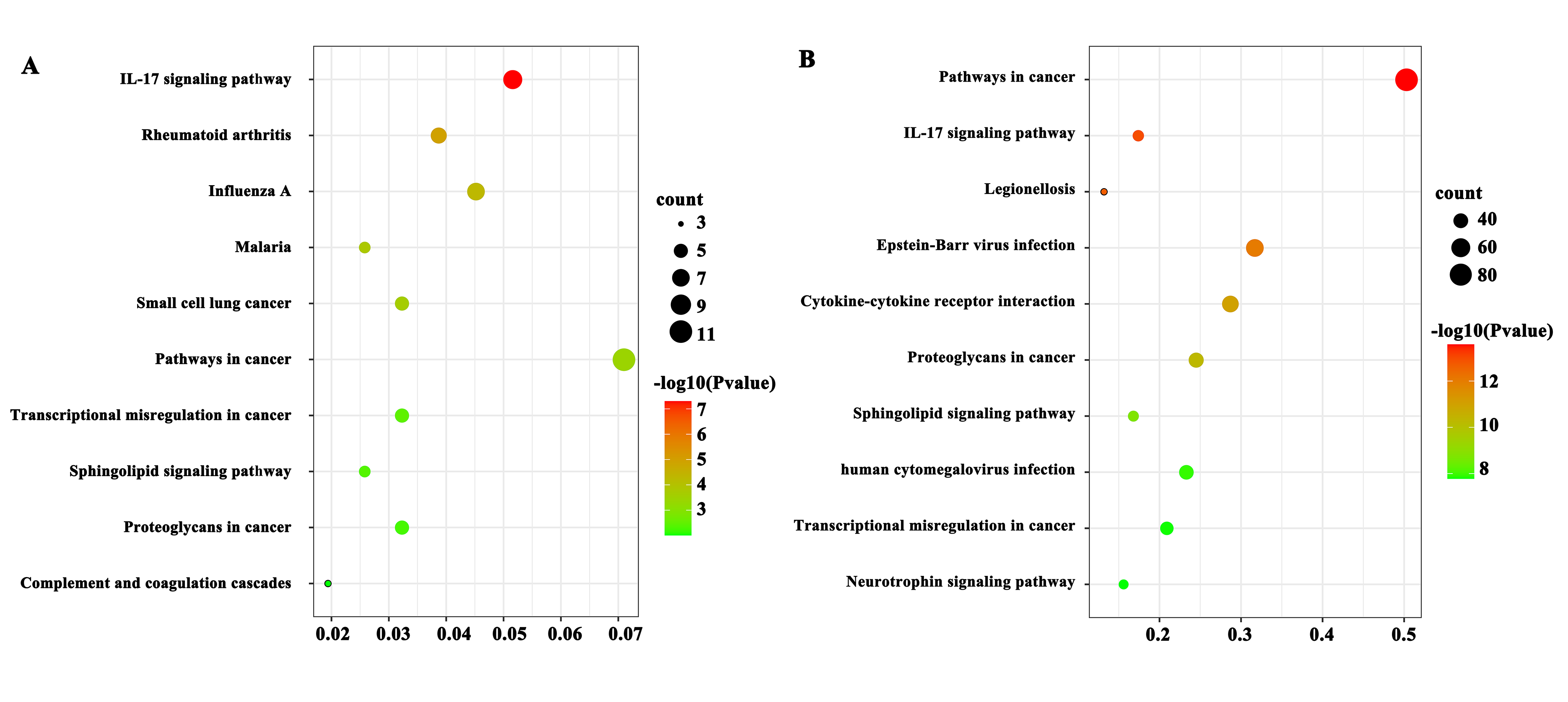

Supplement: Supplementary Figure 5 — KEGG enrichment analysis of DEGs between ICL group vs. NCA group and AMI group vs. NCA group. (A) ICL vs. NCA. (B) AMI vs. NCA. X-axis represents ratios of the number of differential genes to the total gene number in a specific pathway. Y-axis represents different KEGG pathways. The color and size of the dots represent significance and amount of genes enrichment, respectively. DEGs, differentially expressed genes; KEGG, Kyoto Encyclopedia of Genes and Genomes. [file Image_5.TIF]
